# Impaired regenerative capacity and lower revertant fibre expansion in dystrophin-deficient *mdx* muscles on DBA/2 background

**DOI:** 10.1038/srep38371

**Published:** 2016-12-07

**Authors:** Merryl Rodrigues, Yusuke Echigoya, Rika Maruyama, Kenji Rowel Q. Lim, So-ichiro Fukada, Toshifumi Yokota

**Affiliations:** 1 Department of Medical Genetics, University of Alberta Faculty of Medicine and Dentistry, Edmonton, Alberta, T6G2H7, Canada; 2 Laboratory of Molecular and Cellular Physiology, Graduate School of Pharmaceutical Sciences, Osaka University, Suita, Osaka, 565-0871, Japan; 3 Muscular Dystrophy Canada Research Chair, Edmonton, Alberta, T6G2H7, Canada

## Abstract

Duchenne muscular dystrophy, one of the most common lethal genetic disorders, is caused by mutations in the *DMD* gene and a lack of dystrophin protein. In most DMD patients and animal models, sporadic dystrophin-positive muscle fibres, called revertant fibres (RFs), are observed in otherwise dystrophin-negative backgrounds. RFs are thought to arise from skeletal muscle precursor cells and clonally expand with age due to the frequent regeneration of necrotic fibres. Here we examined the effects of genetic background on muscle regeneration and RF expansion by comparing dystrophin-deficient *mdx* mice on the C57BL/6 background (*mdx*-B6) with those on the DBA/2 background (*mdx*-DBA), which have a more severe phenotype. Interestingly, *mdx*-DBA muscles had significantly lower RF expansion than *mdx*-B6 in all age groups, including 2, 6, 12, and 18 months. The percentage of centrally nucleated fibres was also significantly lower in *mdx-*DBA mice compared to *mdx-*B6, indicating that less muscle regeneration occurs in *mdx-*DBA. Our study aligns with the model that RF expansion reflects the activity of precursor cells in skeletal muscles, and it serves as an index of muscle regeneration capacity.

Duchenne muscular dystrophy (DMD) is an X-linked lethal muscular disorder characterized by progressive muscle degeneration, insufficient muscle regeneration, and progressive fibrosis leading to reduced muscle mass^1^. Most DMD patients die between ages 20 to 30 years due to severe respiratory and/or cardiac complications. DMD is caused by frameshift mutations leading to premature stop codons in the *dystrophin (DMD*) gene, which precludes the synthesis of a protein called dystrophin^2^,^3^. Dystrophin, a large (427 kDa) actin-binding protein forms the dystrophin-glycoprotein complex (DGC) at the sarcolemma, in conjunction with dystrophin-associated proteins, such as dystroglycans, neuronal nitric oxide synthase, and sarcoglycans, to name a few^4^,^5^. Dystrophin deficiency perturbs the assembly of DGC and destabilizes the muscle membrane, making it more vulnerable to injury when exposed to stress during muscle contraction or stretch.

Muscles of DMD patients are characterized by the absence of dystrophin protein as represented by Western blotting and immunohistochemistry. In many DMD cases and animal models, however, sporadic dystrophin-positive muscle fibres called revertant fibres (RFs) are observed in otherwise dystrophin-negative backgrounds^6^,^7^,^8^,^9^,^10^,^11^. RFs expand from a subset of skeletal muscle precursor cells (MPCs), called muscle satellite cells, in response to muscle regeneration following degeneration, and are shown either as clusters or as single fibres. The expansion of RFs within a cluster reflects the cumulative history of muscle regeneration and depends on the regenerative capacity of MPCs^7^,^12^. There is no correlation between the number of RFs and the severity of DMD patients, as the RF number ranges between 0.01–7% of myofibres, which is not sufficient to ameliorate symptoms in patients^9^,^13^,^14^. The differences in the number of RFs among DMD patients are more likely caused by their mutation patterns, rather than their muscle regenerative capacity^14^. RFs are also found in dystrophic murine and canine models^6^,^7^,^8^,^12^,^15^. It has been demonstrated in a mouse model that spontaneous exon skipping, which restores a disrupted reading frame by excluding up to 30 exons, leads to the formation of several truncated forms of dystrophin^12^. Although this phenomenon is the basis of exon skipping therapy, which is currently a most promising avenue for treating DMD^16^,^17^,^18^,^19^, the precise mechanisms by which RFs arise are unknown.

*Mdx* (C57BL/10 background, *mdx*-B10), a common dystrophic mouse model, harbors a spontaneous nonsense mutation in exon 23 of the *dystrophin* gene and expresses RFs^20^,^21^,^22^. *Mdx*-B10 mice display a much milder dystrophic phenotype, lower accumulation of fat and fibrosis, and increased skeletal muscle mass for most of their lifespan^20^,^23^,^24^. These differences are likely due to the excellent regenerative capacity of *mdx*-B10 mice and increased expression of utrophin, a dystrophin homolog protein^25^,^26^,^27^,^28^. Fukada and colleagues have developed *mdx* on the DBA/2 background (*mdx*-DBA), a dystrophic mouse model with a more severe phenotype, based on the observation that wild-type DBA/2 mice have more impaired regeneration and loss of muscle weight compared to C57BL/6, BALB/c and C3H/HeN mice following repeated muscle injuries with venom cardiotoxin (CTX)^29^. An *in vitro* assay also revealed that the proliferation ability of isolated satellite cells was significantly lower in DBA/2 mice than in C57BL/6 mice. The prolonged and continuous cycles of degeneration and regeneration result in the exhaustion of satellite cell pools and impaired regeneration of myofibres^30^. These results suggest the lower regenerative capacity of *mdx*-DBA during the course of age-related dystrophic degeneration. Accordingly, expansion of RFs, which are generated from proliferating myogenic cells, is also likely to be lower in *mdx*-DBA than in *mdx*-B6.

To test this hypothesis, we examined muscle regeneration and RF expression/expansion activities during the course of degeneration between *mdx* models with different genetic backgrounds (*mdx*-B6 and *mdx*-DBA/2 mice) from 2 to 18 months of age. The results reveal that *mdx*-DBA muscles have a significantly lower ability in regeneration and RF expansion than *mdx*-B6 across all age groups. Interestingly, the number of both regenerating fibres and single and clustered RFs was consistently lower in *mdx*-DBA by 18 months, while RF expansion in *mdx*-B6 significantly increased with age. The present study demonstrates that genetic backgrounds affect the regenerative capacity of skeletal muscles and the capacity of RF expansion.

## Results

### *Mdx*-DBA displays lower ability to regenerate myofibres than *mdx*-B6 during the course of age-related degeneration

**Figure 1:**
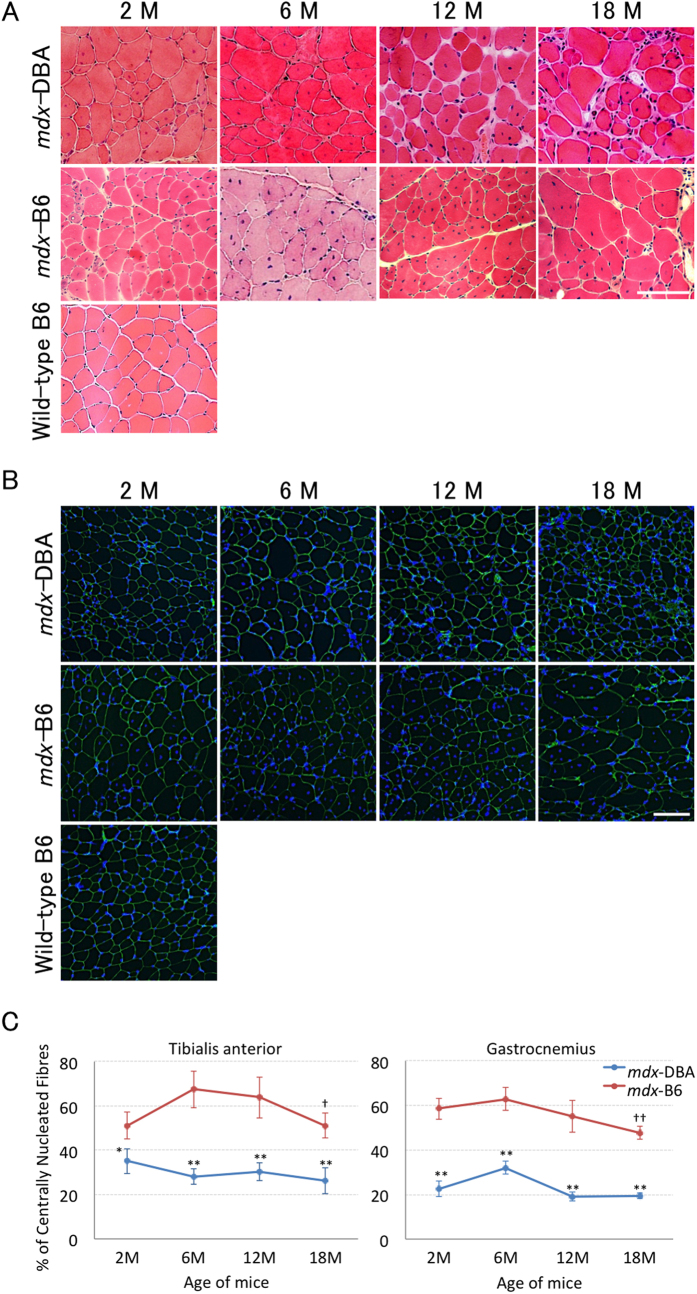
*Mdx*-DBA displays a lower percentage of centrally nucleated fibres than *mdx*-B6. Centrally nucleated fibres were detected by (**A**) haematoxylin and eosin (H&E) staining and (**B**) immunostaining with anti-laminin α2 antibody (green) and DAPI nuclear staining (blue). Representative images in tibialis anterior muscles of *mdx*-DBA and *mdx*-B6 mice at ages 2, 6, 12 and 18 months are shown. Size reduction of myofibres with age was observed in *mdx*-DBA mice. Wild-type C57BL/6 muscle at 2 months of age is shown as a control. M: months. Scale bar = 100 μm. (**C**) The percentage of centrally nucleated fibres per section in H&E staining. Values are represented as mean ± S.D. (n = 4–6 mice per *mdx*-DBA group). H&E staining results of *mdx*-B6 mice were obtained from our previous report with permission^8^. *P < 0.05, **P < 0.01 between *mdx*-DBA and *mdx*-B6 mice; ^†^*P* < 0.05, ^††^*P* < 0.01 compared to 6 months old *mdx*-B6 mice. Statistics: One-Way ANOVA & Tukey-Kramer Multiple Comparisons Test.

To compare the effect of different genetic backgrounds on the long-term regenerative capacity of dystrophic muscles between *mdx*-DBA and *mdx*-B6 mice, we analysed the number of centrally nucleated fibres (CNFs) and developmental myosin heavy chain (MHCd)-positive myofibres, indicative of cumulative and current muscle regeneration, respectively, at 2, 6, 12, and 18 months of age. CNFs in tibialis anterior (TA) and gastrocnemius (GC) muscles were detected with haematoxylin and eosin (H&E) staining. The nuclei of newly regenerated muscle fibres are centrally located while those of mature muscle fibres are peripherally located. Dystrophic muscles of *mdx*-B10 and *mdx*-B6 display persistent and an overwhelming number of CNFs due to continuous regeneration after muscle degeneration from the age of around 2 months. Here we found that *mdx*-DBA mice showed a significantly lower percentage of CNFs than *mdx*-B6 mice in TA and GC muscles in all age groups (Fig. 1A,B,C). The percentage of CNFs peaked at 6 months of age and then showed a downward decline with a significant decrease at 18 months (compared to 6 months) in TA and GC muscles of *mdx*-B6 mice (Fig. 1C). In contrast, the percentage of CNFs in *mdx*-DBA mice showed no statistically significant differences between age groups in both TA and GC muscles. The consistency of the results obtained with H&E staining was confirmed with immunohistochemistry using anti-laminin α2 antibody (as a sarcolemmal marker) and DAPI nuclear staining (Fig. 1B). Marked size reduction of myofibres (atrophy) was observed in both TA and GC muscles of *mdx*-DBA at the age of 18 months compared to 2 months.

**Figure 2:**
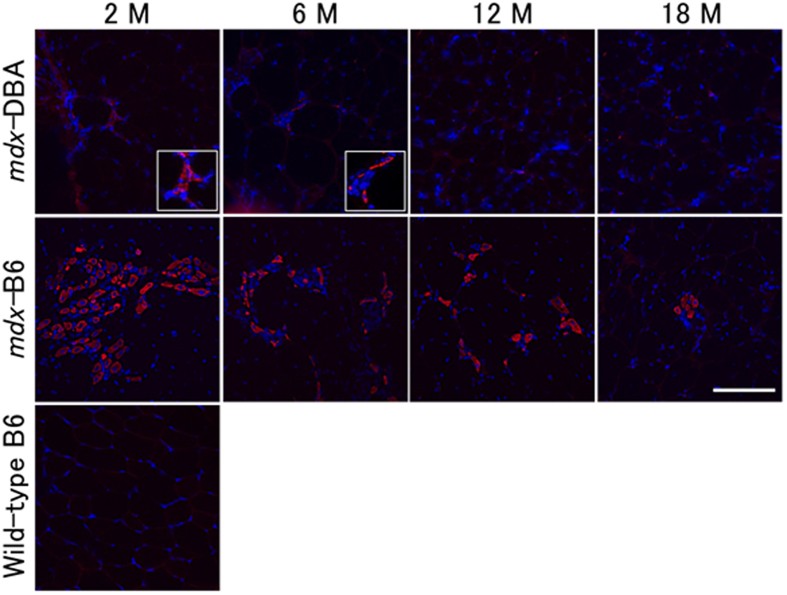
Nascent regenerating muscle fibres are rarely observed in *mdx*-DBA. The regenerating fibres in tibialis anterior muscles were detected with anti-developmental myosin heavy chain (MHCd) antibody (red). Nuclei were stained with DAPI (blue). Insets depict zoomed-in MHCd-positive fibres defined by the white box. MHCd-positive fibres were not detected in *mdx*-DBA at 12 and 18 months of age. Representative images are shown from 4–6 mice in each age group. Wild-type C57BL/6 TA muscle at 2 months old is shown as a negative control. M: months. Scale bar = 100 μm.

We also analysed nascent regenerating myofibres with immunohistochemistry using anti-MHCd antibody in *mdx*-DBA and *mdx*-B6. MHCd-positive fibres on severely affected areas independent of age, in which myofibres were replaced with fibrous connective tissues, were excluded from the counting. In *mdx*-DBA, MHCd-positive fibres were barely detectable across all ages (Fig. 2). Only an average of 1 to 2 MHCd-positive fibres per section was detected in TA muscles of *mdx*-DBA mice at 2 and 6 months of age, and they were not observed in older *mdx*-DBA at 12 and 18 months of age. In contrast, more MHCd-positive regenerating fibres were found in all age groups of *mdx*-B6, and the number was decreased with age: 154, 98, 97, and 62 positive fibres on average at 2, 6, 12, and 18 months, respectively. These results suggest that muscle regeneration is more active in *mdx-*B6 than *mdx-*DBA overall.

### Necrotic myofibres and accumulation of immunoglobulin in myofibre periphery in *mdx*-DBA and *mdx*-B6 muscles

**Figure 3:**
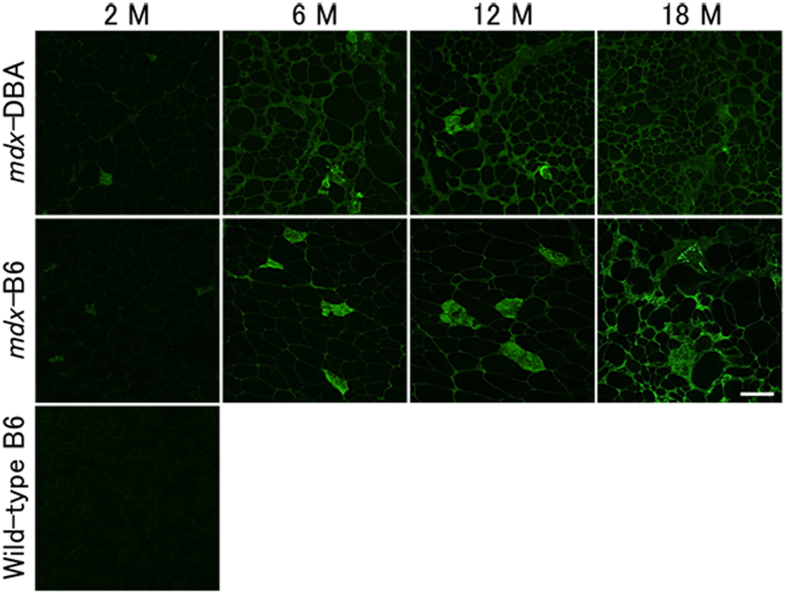
Immunoglobulin accumulation in *mdx*-DBA muscles and *mdx*-B6 muscles. The accumulation of immunoglobulin in tibialis anterior and gastrocnemius muscles was detected by anti-mouse IgG antibody. IgG-positive fibres represent necrotic fibres. Representative images in tibialis anterior muscles are shown from 4–7 mice in each age group of *mdx*-DBA and *mdx*-B6. Wild-type C57BL/6 TA muscle at 2 months old is shown as a negative control. M: months. Scale bar = 100 μm.

In dystrophic muscles of DMD patients and mouse models, necrotic muscle fibres stain positively for immunoglobulins such as IgG and IgM^31^,^32^. To compare necrosis between *mdx-*B6 and *mdx-*DBA muscles, we employed immunohistochemistry with anti-mouse IgG antibody. IgG-positive necrotic fibres were analysed in TA and GC muscles of *mdx*-DBA and *mdx*-B6 mice. We observed very few IgG-positive necrotic fibres in both *mdx*-DBA mice and *mdx*-B6 mice at 2–18 months of age (Fig. 3). IgG signals surrounding individual myofibres were observed from 6 months of age in *mdx*-DBA and from 12 months in *mdx*-B6. Overall, we did not observe significant differences in the number of necrotic fibres between *mdx-*B6 and *mdx-*DBA.

### Expression and expansion of revertant fibres (RFs) are lower in *mdx*-DBA mice compared to *mdx*-B6 mice

**Figure 4:**
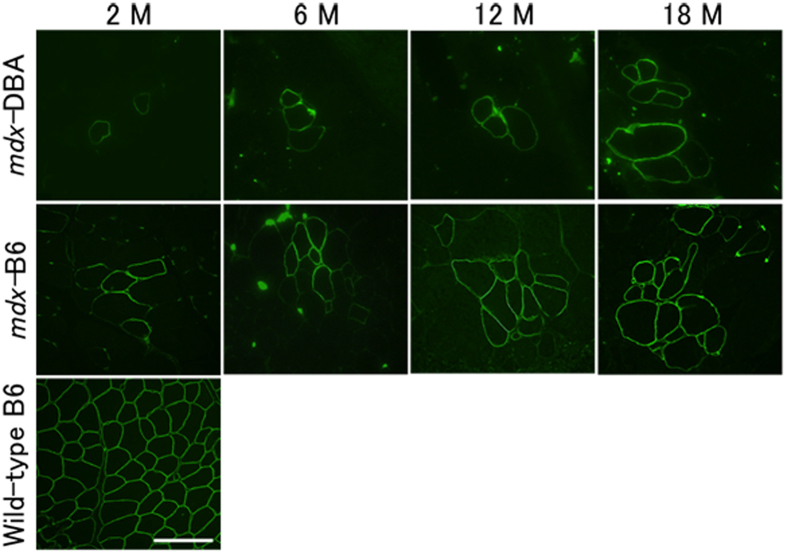
Dystrophin-positive revertant fibres (RFs) at ages 2, 6, 12, and 18 months in *mdx*-DBA and *mdx*-B6 mice. Dystrophin signals were detected with an anti-dystrophin C-terminal antibody. Representative images of the maximum RFs in a cluster in tibialis anterior muscles are shown from 3–6 mice in each age group of *mdx*-DBA and *mdx*-B6. *Mdx*-B6 shows higher RF expansion than *mdx*-DBA in all age groups. Wild-type C57BL/6 TA muscle at 2 months old is shown as a positive control. M: months. Scale bar = 100 μm.

**Figure 5:**
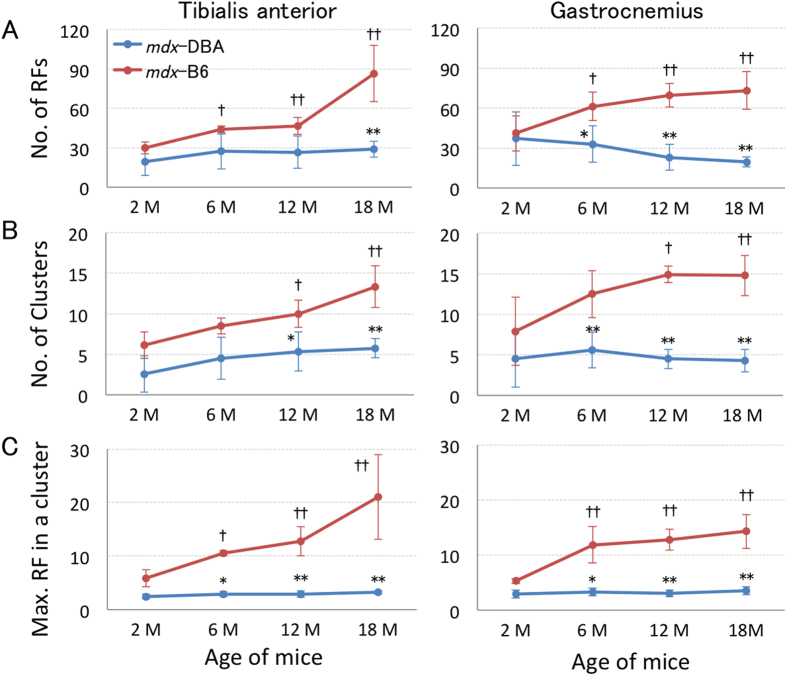
Expression and expansion of RFs are lower in *mdx*-DBA mice compared to *mdx*-B6 mice in tibialis anterior and gastrocnemius muscles at 2, 6, 12 and 18 months of age. The number of RFs per section was counted according to the following categories: (**A**) the number of RFs, (**B**) the number of RF clusters, and (**C**) the maximum number of RFs in a single cluster. *Mdx*-B6 mice have a significantly higher RF expression in all categories than *mdx*-DBA mice. Values are scored as mean ± S.D. (n = 4–6 *mdx*-DBA mice per each group). Data for *mdx*-B6 mice were obtained from our previous report with permission^8^. *P < 0.05, **P < 0.01 between *mdx*-DBA and *mdx*-B6 mice; ^†^*P* < 0.05, ^††^*P* < 0.05 compared to 2 months old *mdx*-B6 mice. M: months. Statistics: One-Way ANOVA & Tukey-Kramer Multiple Comparisons Test.

Next, we examined if RF expression and expansion are changed in a mouse model with the same mutation, but with different genetic backgrounds (DBA/2 and C57BL/6). An identical methodology of immunohistochemistry was employed for both mouse models (*mdx*-DBA and *mdx*-B6)^8^. An anti-dystrophin C-terminal domain antibody was used to detect RFs as the C-terminal domain is highly conserved in most truncated dystrophin proteins^7^,^12^,^33^. RFs were categorized as A) the number of RFs, B) the number of RF clusters and C) the maximum RF number in a cluster. Although RFs were observed in *mdx*-DBA and *mdx*-B6 of all ages (Fig. 4), the total RF number per section was significantly lower in *mdx*-DBA at 12 months and 18 months compared to *mdx*-B6 (Fig. 5A). Unlike *mdx*-B6 mice, *mdx*-DBA mice did not exhibit an increase in the number of RF clusters and RF cluster size (the maximum RF number per cluster) over time between ages 2 and 18 months (Fig. 5B,C). While *mdx*-B6 mice showed an age-related increase across all measured categories, no age-related change was observed in *mdx*-DBA in both TA and GC muscles. These data reveal that *mdx*-DBA mice have a lower capacity for RF generation regardless of age compared to *mdx*-B6, and the accumulation of RFs over time was not found in *mdx* mice on the DBA/2 background.

## Discussion

RFs are observed in DMD patients and animal models and have been reported to expand with age in dystrophic mouse models^7^,^8^,^12^. The mechanism behind the expression of RFs might hold clues for developing novel therapeutic applications for DMD, but it is poorly understood. It is hypothesized that when myofibres surrounding the area around an RF degenerate, revertant satellite stem cells encoding an altered splicing pattern for dystrophin expression become activated during the regeneration process and cause the clonal expansion of RFs. Yokota *et al*. have shown that irradiated muscles (consisting of damaged muscle stem cells) of *mdx*-B10 mice cannot regenerate after continuous degeneration and are consequently unable to display RF expansion^7^. There seems to be a negative relationship between stem cell depletion and expansion of RFs. We previously reported distinct RF expression and expansion in mice with a nonsense mutation in exon 23 (*mdx*) versus exon 52 deletion mutation (*mdx52*) on the same C57BL/6 background^8^. Age-related muscle regeneration capacity is also different between these mouse models. These studies suggest that RF expansion is associated with continuous regeneration of dystrophic muscles, age, and mutation type. Here we compared *mdx* mice with the same mutation (a nonsense mutation in exon 23) but different genetic backgrounds, C57BL/6 and DBA/2, and revealed the distinct regenerative capacity and RF generation between them.

Coley *et al*., have recently reported that *mdx*-DBA exhibits impaired skeletal and cardiac muscle functions at an earlier age than *mdx*-B10^34^. We also previously reported that compared to *mdx*-B10, *mdx*-DBA mice showed the greater decrease in skeletal muscle function and weight, and increased accumulation of fat and fibrosis, but did not show increased muscle degeneration^29^. In the present study, we found that *mdx*-DBA mice have fewer CNFs and nascent regenerating myofibres across all age groups, suggesting a lower capacity to regenerate myofibres than *mdx*-B6 mice. Hence, the more severe phenotype of *mdx*-DBA mice may be attributable to the reduced muscle regeneration ability. The milder phenotypes associated with *mdx*-B6 and *mdx*-B10 mouse models could be partly attributable to the upregulation of utrophin (a dystrophin homolog) at the sarcolemma, which compensates for the lack of dystrophin^25^,^26^. Although utrophin levels remain to be determined in the present study, our results suggest that a notable impairment in muscle regeneration (impaired satellite cell potential), as seen in *mdx*-DBA, could lead to more severe muscle phenotypes than *mdx* on other backgrounds.

The present results demonstrated that RF expression was lower in *mdx*-DBA mice compared to *mdx*-B6 mice. The number of RF clusters (reflects the frequency of RF generation) and the maximum number of RFs in a cluster (reflects the clonal expansion of RFs) were also lower in *mdx*-DBA at later ages. Because RF generation is largely dependent on the function and the number of satellite cells (MPCs)^7^, the absence of the age-related increase in RFs in *mdx*-DBA suggests that the MPCs from the *mdx-*DBA have a lower proliferation or differentiation capacity when compared to *mdx*-B6. We have previously reported that the satellite cells of wild-type DBA/2 mice at 6-week-old showed lower bromodeoxyuridine (BrdU) uptake of myoblasts (lower proliferation of satellite cells) than C57BL/6 mice. Our results showed no statistically significant difference in RF expression at 2 months of age between *mdx*-DBA and *mdx*-B6 mice. However, an incremental difference in RF expression between the two models was observed from 6 to 18 months of age. This suggests that there is no notable difference in initial regeneration ability between *mdx*-B6 and *mdx-*DBA, but that the long-term regeneration ability of satellite cells in *mdx-*DBA is compromised after continuous muscle degeneration. We previously revealed that DBA/2 muscles showed almost the same ability to regenerate as C57BL/6 when injured by CTX only once, however, they showed the greater impairment of regeneration when injured repeatedly, which might be attributable to more reduced activity of self-renewal in muscle satellite cells of DBA/2 mice^29^.

Taken together, the present study demonstrated that DBA/2 genetic background contributes to lower capacity to generate and clonally expand RFs in *mdx*. This suggests that *mdx*-DBA could become a more appropriate model for assessing the effect of therapies (such as exon skipping) since the dystrophin levels attributed to the effects of therapies are less confounded by dystrophin-positive revertant fibres. Our results align with previous studies showing that RF expansion is associated with the regenerative capacity of skeletal muscle. Currently, several dystrophic mouse models with different mutations and severity (e.g. *mdx52*) are available^22^,^24^. As observed in *mdx*-DBA, these mouse models backcrossed into DBA/2 mice may also be helpful for better understanding of dystrophic pathology.

## Methods

### Murine Models

DBA/2 mice at six-week-old were obtained from Charles River Japan (Yokohama, Japan). *Mdx* mice with C57BL/10 background were prepared from Central Laboratories of Experimental Animals (Kanagawa, Japan), and were backcrossed into mice with a DBA/2 background. Affected male and female (homozygous mutant) *mdx*-DBA mice at the 10th generation were used in this study. A point mutation in exon 23 in the *mdx* mice was confirmed by genotyping as previously described^35^. Mice at 2, 6, 12, and 18 months of age were euthanized by cervical dislocation. *Mdx*-B6 and control C57BL/6 mice were prepared as previously described^8^. The Experimental Animal Care and Use Committee at Osaka University, National Center of Neurology and Psychiatry, and the University of Alberta approved all procedures for experimental animals. Experiments were conducted in accordance with the approved guidelines.

### Preparation of muscle samples

Tibialis anterior and gastrocnemius muscles were collected immediately after euthanising mice. Muscle tissues were put in tragacanth gum on a cork stage, frozen in liquid nitrogen-cooled isopentane, and then stored at −80 °C^6^. Transverse cryosections of TA and GC muscles (7-μm thickness) were made with LEICA CM1950 cryostat (Wetzlar, Germany). Sections were put on poly-L-lysine coated slide glasses, dried for at least 30 minutes and then stored at −80 °C for later use. Frozen muscle sections were air-dried for 30 minutes at room temperature prior to subsequent staining.

### Haematoxylin and eosin staining

Haematoxylin and eosin (H&E) staining was conducted with Mayer’s haematoxylin and eosin solutions as described in our previous study^8^. Samples were visualised under bright field illumination using a 20x objective lens in Nikon Eclipse TE 2000-U (Nikon, Tokyo, Japan). Four to eight images were randomly taken from individual sections of a muscle sample for analysing the percentage of centrally nucleated fibres. Approximately 500 to 1000 of the total number of myofibres (normal and centrally nucleated fibres) were counted in each muscle tissue of *mdx*-DBA mice. The percentage of CNFs was calculated with the ratio of the number of CNFs to that of the total fibres. Data regarding *mdx*-B6 mice were obtained from our previous report^8^.

### Immunohistochemistry

In RF analysis, muscle sections were incubated with a blocking solution consisting of phosphate-buffered saline (PBS) with 10% goat serum and 0.1% TritonX-100 for 20 minutes at room temperature. Next, the sections were incubated with a rabbit polyclonal primary antibody (1:400 in the blocking solution) against human dystrophin C-terminal (position at 3,661–3,677 amino acids; Abcam, Bristol, UK) overnight at 4 °C. After washing the sections with PBS three times, the sections were incubated with AlexaFluor^TM^ 488-conjugated goat anti-rabbit IgG H+L secondary antibody (1:2,000 in PBS; Molecular Probes, OR, USA) for an hour at room temperature. For CNF analysis, muscle sections were incubated with rat anti-laminin α2 monoclonal antibody (1:100 in PBS with 0.1% Triton-X 100, ALX-804-190, Enzo Life Sciences, NY, USA) for an hour at room temperature followed by the incubation with DyLight 488-condjugated goat anti-rat IgG H+L antibody (1:250, Thermo Fisher Scientific, CA, USA) for an hour at room temperature. To detect nascent regenerating fibres, mouse anti-myosin developmental type heavy chain antibody (1:20, NCL-MHCd, Leica Biosystems, Newcastle, UK) was used with VECTOR^®^ M.O.M.^TM^ Immunodetection Kit BASIC (Vector Laboratories, Peterborough, UK). Immunoglobulin-accumulated necrotic myofibres were detected with AlexaFluor^TM^ 488-conjugated goat anti-mouse IgG F(ab’)_2_ antibody (1:750 in PBS with 0.1% Triton-X 100, Molecular Probes) after blocking with PBS containing 10% goat serum and 0.1% Triton-X for 20 min at room temperature, as previously described^32^. Nuclear counterstaining was performed with 4′,6-diamidino-2-phenylindole (DAPI) in a mounting agent (Vectashield; Vector Laboratories, CA, USA). Signals were detected with a fluorescence microscope (Nikon Eclipse TE 2000-U).

### Assessment of RFs and nascent regenerating fibres

The number of RFs and its clusters, and the maximum number of RFs in a cluster were scored in TA and GC muscles of mice at the age of 2, 6, 12, and 18 months. A dystrophin-positive RF was scored when more than half of its membrane circumference expressed a green positive signal. A single cluster is comprised of RFs immediately adjacent to each other. The number of RFs and its clusters was averaged with four sections at intervals of more than 100 μm from the muscle belly in individual muscle samples. Data for *mdx*-B6 were adapted from our previous results with permission^8^. In the analysis of nascent regenerating myofibres detected with anti-developmental MHC antibody, the highest number in three serial sections at 100 μm intervals was averaged with individual mouse data. Clusters of MHCd-positive fibres that arose due to severe degeneration independent of age were excluded from counting.

### Statistical analysis

Numerical results were expressed as mean values ± standard deviation (S.D.) for each age group. Statistical significance was assessed by one-way ANOVA followed by Tukey–Kramer multiple comparison test. A probability less than 5% (*P* < 0.05) or less than 1% (*P* < 0.01) was considered statistically significant.

## Additional Information

**How to cite this article**: Rodrigues, M. *et al*. Impaired regenerative capacity and lower revertant fibre expansion in dystrophin-deficient *mdx* muscles on DBA/2 background. *Sci. Rep.* **6**, 38371; doi: 10.1038/srep38371 (2016).

**Publisher's note:** Springer Nature remains neutral with regard to jurisdictional claims in published maps and institutional affiliations.

## References

[b1] Flanigan K. M. Duchenne and Becker Muscular Dystrophies. Neurologic clinics 32, 671–688, doi: 10.1016/j.ncl.2014.05.002 (2014). 25037084

[b2] Koenig M. . Complete cloning of the Duchenne muscular dystrophy (DMD) cDNA and preliminary genomic organization of the DMD gene in normal and affected individuals. Cell 50, 509–517 (1987). 3607877 10.1016/0092-8674(87)90504-6

[b3] Hoffman E. P., Brown R. H. Jr. & Kunkel L. M. Dystrophin: the protein product of the Duchenne muscular dystrophy locus. Cell 51, 919–928 (1987). 3319190 10.1016/0092-8674(87)90579-4

[b4] Ervasti J. M. Dystrophin, its interactions with other proteins, and implications for muscular dystrophy. Biochimica et biophysica acta 1772, 108–117, doi: 10.1016/j.bbadis.2006.05.010 (2007). 16829057

[b5] Nichols B., Takeda S. & Yokota T. Nonmechanical Roles of Dystrophin and Associated Proteins in Exercise, Neuromuscular Junctions, and Brains. Brain sciences 5, 275–298, doi: 10.3390/brainsci5030275 (2015). 26230713 PMC4588140

[b6] Yokota T., Hoffman E. & Takeda S. Antisense oligo-mediated multiple exon skipping in a dog model of duchenne muscular dystrophy. Methods in molecular biology 709, 299–312, doi: 10.1007/978-1-61737-982-6_20 (2011). 21194037 PMC4491489

[b7] Yokota T. . Expansion of revertant fibers in dystrophic mdx muscles reflects activity of muscle precursor cells and serves as an index of muscle regeneration. J Cell Sci 119, 2679–2687, doi: 10.1242/jcs.03000 (2006). 16757519

[b8] Echigoya Y. . Mutation types and aging differently affect revertant fiber expansion in dystrophic mdx and mdx52 mice. PloS one 8, e69194, doi: 10.1371/journal.pone.0069194 (2013). 23894429 PMC3722172

[b9] Nicholson L. V., Johnson M. A., Gardner-Medwin D., Bhattacharya S. & Harris J. B. Heterogeneity of dystrophin expression in patients with Duchenne and Becker muscular dystrophy. Acta neuropathologica 80, 239–250 (1990). 2205076 10.1007/BF00294640

[b10] Nicholson L. V. . Dystrophin in skeletal muscle. II. Immunoreactivity in patients with Xp21 muscular dystrophy. J Neurol Sci 94, 137–146 (1989). 2693618 10.1016/0022-510x(89)90224-4

[b11] Klein C. J. . Somatic reversion/suppression in Duchenne muscular dystrophy (DMD): evidence supporting a frame-restoring mechanism in rare dystrophin-positive fibers. American journal of human genetics 50, 950–959 (1992). 1570844 PMC1682584

[b12] Lu Q. L. . Massive idiosyncratic exon skipping corrects the nonsense mutation in dystrophic mouse muscle and produces functional revertant fibers by clonal expansion. The Journal of cell biology 148, 985–996 (2000). 10704448 10.1083/jcb.148.5.985 PMC2174546

[b13] Fanin M., Danieli G. A., Vitiello L., Senter L. & Angelini C. Prevalence of dystrophin-positive fibers in 85 Duchenne muscular dystrophy patients. Neuromuscular disorders: NMD 2, 41–45 (1992). 1525557 10.1016/0960-8966(92)90025-2

[b14] Arechavala-Gomeza V. . Revertant fibres and dystrophin traces in Duchenne muscular dystrophy: implication for clinical trials. Neuromuscular disorders: NMD 20, 295–301, doi: 10.1016/j.nmd.2010.03.007 (2010). 20395141

[b15] Hoffman E. P., Morgan J. E., Watkins S. C. & Partridge T. A. Somatic reversion/suppression of the mouse mdx phenotype *in vivo*. J Neurol Sci 99, 9–25 (1990). 2250176 10.1016/0022-510x(90)90195-s

[b16] Yokota T. . Efficacy of systemic morpholino exon-skipping in Duchenne dystrophy dogs. Annals of neurology 65, 667–676, doi: 10.1002/ana.21627 (2009). 19288467 PMC5951302

[b17] Lu Q. L. . Systemic delivery of antisense oligoribonucleotide restores dystrophin expression in body-wide skeletal muscles. Proceedings of the National Academy of Sciences of the United States of America 102, 198–203, doi: 10.1073/pnas.0406700102 (2005). 15608067 PMC544058

[b18] Mendell J. R. . Longitudinal effect of eteplirsen versus historical control on ambulation in Duchenne muscular dystrophy. Annals of neurology 79, 257–271, doi: 10.1002/ana.24555 (2016). 26573217 PMC5064753

[b19] Lee J. & Yokota T. Antisense Therapy in Neurology. Journal of Personalized Medicine 3, 144–176, doi: 10.3390/jpm3030144 (2013). 25562650 PMC4251390

[b20] Stedman H. H. . The mdx mouse diaphragm reproduces the degenerative changes of Duchenne muscular dystrophy. Nature 352, 536–539, doi: 10.1038/352536a0 (1991). 1865908

[b21] Sicinski P. . The molecular basis of muscular dystrophy in the mdx mouse: a point mutation. Science (New York, N.Y.) 244, 1578–1580 (1989). 10.1126/science.2662404 2662404

[b22] Rodrigues M., Echigoya Y., Fukada S. & Yokota T. Current Translational Research and Murine Models For Duchenne Muscular Dystrophy. J Neuromuscul Dis 3, 29–48, doi: 10.3233/JND-150113 (2016). 27854202 PMC5271422

[b23] Grounds M. D., Radley H. G., Lynch G. S., Nagaraju K. & De Luca A. Towards developing standard operating procedures for pre-clinical testing in the mdx mouse model of Duchenne muscular dystrophy. Neurobiology of disease 31, 1–19, doi: 10.1016/j.nbd.2008.03.008 (2008). 18499465 PMC2518169

[b24] Willmann R., Possekel S., Dubach-Powell J., Meier T. & Ruegg M. A. Mammalian animal models for Duchenne muscular dystrophy. Neuromuscular disorders: NMD 19, 241–249, doi: 10.1016/j.nmd.2008.11.015 (2009). 19217290

[b25] Matsumura K., Ervasti J. M., Ohlendieck K., Kahl S. D. & Campbell K. P. Association of dystrophin-related protein with dystrophin-associated proteins in mdx mouse muscle. Nature 360, 588–591, doi: 10.1038/360588a0 (1992). 1461282

[b26] Law D. J., Allen D. L. & Tidball J. G. Talin, vinculin and DRP (utrophin) concentrations are increased at mdx myotendinous junctions following onset of necrosis. J Cell Sci 107 (Pt 6), 1477–1483 (1994). 7962191 10.1242/jcs.107.6.1477

[b27] Deconinck A. E. . Utrophin-dystrophin-deficient mice as a model for Duchenne muscular dystrophy. Cell 90, 717–727 (1997). 9288751 10.1016/s0092-8674(00)80532-2

[b28] Grady R. M. . Skeletal and cardiac myopathies in mice lacking utrophin and dystrophin: a model for Duchenne muscular dystrophy. Cell 90, 729–738 (1997). 9288752 10.1016/s0092-8674(00)80533-4

[b29] Fukada S. . Genetic background affects properties of satellite cells and mdx phenotypes. The American journal of pathology 176, 2414–2424, doi: 10.2353/ajpath.2010.090887 (2010). 20304955 PMC2861106

[b30] Luz M. A., Marques M. J. & Santo Neto H. Impaired regeneration of dystrophin-deficient muscle fibers is caused by exhaustion of myogenic cells. Brazilian journal of medical and biological research = Revista brasileira de pesquisas medicas e biologicas/Sociedade Brasileira de Biofisica … [et al.] 35, 691–695 (2002). 10.1590/s0100-879x2002000600009 12045834

[b31] Straub V., Rafael J. A., Chamberlain J. S. & Campbell K. P. Animal models for muscular dystrophy show different patterns of sarcolemmal disruption. The Journal of cell biology 139, 375–385 (1997). 9334342 10.1083/jcb.139.2.375 PMC2139791

[b32] Echigoya Y. . Long-term efficacy of systemic multiexon skipping targeting dystrophin exons 45-55 with a cocktail of vivo-morpholinos in mdx52 mice. Molecular therapy. Nucleic acids 4, e225, doi: 10.1038/mtna.2014.76 (2015). 25647512 PMC4345310

[b33] Winnard A. V., Mendell J. R., Prior T. W., Florence J. & Burghes A. H. Frameshift deletions of exons 3-7 and revertant fibers in Duchenne muscular dystrophy: mechanisms of dystrophin production. American journal of human genetics 56, 158–166 (1995). 7825572 PMC1801338

[b34] Coley W. D. . Effect of genetic background on the dystrophic phenotype in mdx mice. Hum Mol Genet 25, 130–145, doi: 10.1093/hmg/ddv460 (2016). 26566673 PMC4690497

[b35] Amalfitano A. & Chamberlain J. S. The mdx-amplification-resistant mutation system assay, a simple and rapid polymerase chain reaction-based detection of the mdx allele. Muscle & nerve 19, 1549–1553, doi: 10.1002/(SICI)1097-4598(199612)19:12&lt;1549::AID-MUS4&gt;3.0.CO;2-A (1996). 8941268

